# Investigating PEDOT:PSS Binder as an Energy Extender in Sulfur Cathodes for Li–S Batteries

**DOI:** 10.1021/acsaem.4c01553

**Published:** 2024-08-26

**Authors:** Matthew Dent, Sean Grabe, Obehi Ayere, Shumaila Babar, Mateus G. Masteghin, David C. Cox, Brendan J. Howlin, Mark A. Baker, Constantina Lekakou

**Affiliations:** † Center for Engineering Materials, School of Mechanical Engineering Sciences, University of Surrey, Guildford, Surrey GU2 7XH, U.K.; ‡ Advanced Technology Institute, University of Surrey, Guildford, Surrey GU2 7XH, U.K.; § School of Chemistry and Chemical Engineering, University of Surrey, Guildford, Surrey GU2 7XH, U.K.

**Keywords:** lithium–sulfur batteries, PEDOT:PSS, supercapacitor, multiscale modeling, polysulfide migration, surface redox

## Abstract

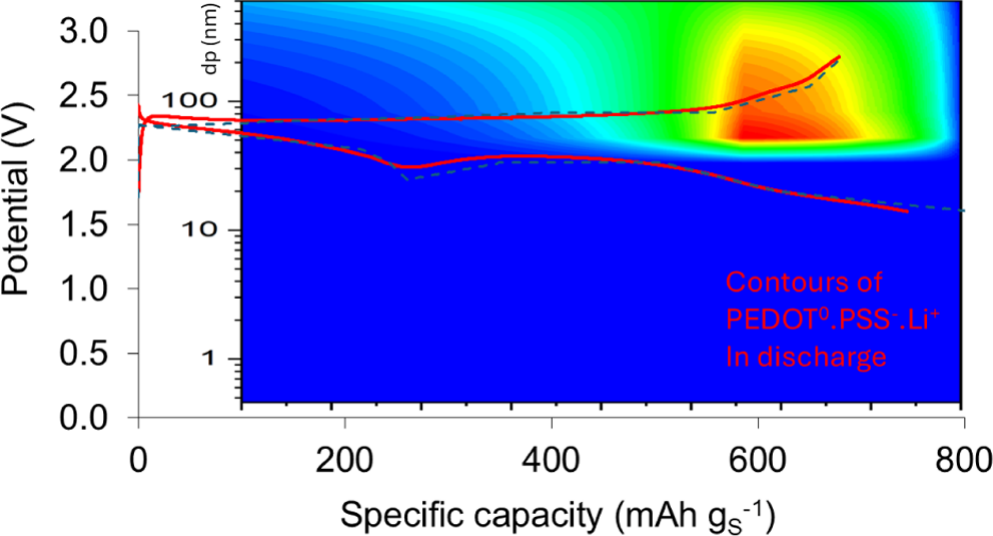

Although lithium–sulfur (Li–S) batteries offer a high theoretical energy density, shuttling of dissolved sulfur and polysulfides is a major factor limiting the specific capacity, energy density, and cyclability of Li–S batteries with a liquid electrolyte. Cathode host materials with a microstructure to restrict the migration of active material may not totally eliminate the shuttling effect or may create additional problems that limit the full dissolution and redox conversion of all active cathode materials. Selecting a cathode coating binder with a multifunctional role offers a universal solution suitable for various cathode hosts. PEDOT:PSS is investigated as such a binder in this study via experimental testing and material characterization as well as multiscale modeling. The study is based on Li–S cells with a sulfur cathode in hollow porous particles as the cathode host and the 10 wt % PEDOT:PSS binder and electrolyte 1 M LiTFSI in 1:1 DOL:DME 1:1 v/v. A reference supercapacitor cell with the same electrolyte and electrodes comprising a coating of the same hollow porous particles and 10 wt % PEDOT:PSS revealed the pseudocapacitive effect of PEDOT:PSS following a surface redox mechanism that dominates the charge phase, which is equivalent to the discharge phase of the Li–S battery cell. A multipore continuum model for supercapacitors and Li–S cells is extended to incorporate the pseudocapacitive effects of PEDOT:PSS with the Li^+^ ions and the adsorption effects of PEDOT:PSS with respect to sulfur and lithium sulfides in Li–S cells, with the adsorption energies determined via molecular and *ab initio* simulations in this study. Experimental data and predictions of multiscale simulations concluded a 7–9% extension of the specific capacity of Li–S battery cells due to the surface redox effect of PEDOT:PSS and elimination of lithium sulfides from the anode by slowing down their migration and shuttling via their adsorption by the PEDOT:PSS binder.

## 1 Introduction

Energy density and cost remain key factors in battery selection for many sectors, such as batteries for transport applications and portable devices. Under such criteria, lithium–sulfur (Li–S) batteries offer a high theoretical energy density of 2510 Wh kg^–1^ and a low-cost sulfur cathode consisting of abundant materials compared to the various types of cathodes of Li-ion batteries containing a substantial proportion of critical raw materials (CRMs), which raise the cost.^1−3^ The insulating nature of sulfur and its expansion up to 70% if fully converted to Li_2_S at the end of discharge^4^ have guided the use and development of conductive porous hosts that may accommodate up to 70 wt % sulfur in the cathode and its expansion.^5^ Such materials include activated carbon (AC) fabrics,^6−8^ AC coatings,^9,10^ graphene composites with other conductive nanoadditives^11,12^ and carbon nanotube networks^13^ or aligned structures.^14,15^ Experimental^16,17^ and computational studies^7,18,19^ of sulfur cathodes have detected two important challenges to be overcome for realizing commercialization of Li–S batteries: (a) the “shuttling” of soluble sulfides and sulfur, starting with their migration to the anode during discharge and continuous “shuttling” between anode and cathode during charge and (b) slow redox conversion in undissolved sulfur and sulfides, especially in small micropores, due to the low rate of Li^+^ ion migration in the solid state. Both of these problems result in underutilization of active material and reduction of specific capacity.

Hollow porous carbon particle-based coatings have been proposed as cathode hosts to trap sulfur and sulfides^20,21^ with some improvement in limiting the “shuttling” of polysulfides but not total elimination of the problem.^18^ Functionalization of the porous hollow particle hosts with single atom catalysts (SACs) that trap sulfur and sulfides via increased adsorption energy^22^ may help in further reducing the problem,^23^ but the amount of functional groups and their positioning needs to be carefully controlled as it reduces the pore size of the host, may block Li^+^ ion migration, and hence block the utilization of active material leading to a decrease of specific capacity.^24^ Furthermore, the SACs may involve CRMs^22,23^ and laborious, energy-consuming processes for the host functionalization.^23,25−27^

A valid alternative investigated in this study is the use of a suitable binder in the coating of porous hollow particle hosts impregnated with sulfur. PEDOT:PSS is a conducting polymer with high transverse and in-plane conductivity for inkjet printed films^28^ and high in-plane conductivity for spin-coated films.^29,30^ Use as a binder in AC-based electrode coatings for supercapacitors with the Li-ion electrolyte raised the specific capacitance in galvanostatic discharge by 15% via a surface redox mechanism and 19% via combined intercalation and redox mechanisms for electrodes fabricated by the doctor blade technique and spraying, respectively.^31^ The latter exhibited small plateaus at 1.4–1.6 V in charge (Li^+^ ion intercalation) and 0.4–0.7 V in discharge (Li^+^ ion deintercalation).^31^ Although any prior use of PEDOT:PSS as a binder in sulfur cathodes was thought to be beneficial for Li–S batteries,^32,33^ the mechanisms of the contribution were not elucidated. Other conductive and pseudocapacitive polymers such as polyaniline (PANI) have been used in the cathode of Li–S batteries in the form of carbon/PANI composites but with a different binder such as poly(vinylidene difluoride) (PVDF).^34,35^ The reason for not using PANI as a binder is that synthesis steps are required for PANI from a liquid-feeding mixture containing an aniline monomer, which is a toxic compound that needs to be processed in an air-free atmosphere to avoid side reactions with aniline and parasitic byproducts. Incorporating the PANI synthesis step would be difficult in the cathode coating fabrication on a large scale. As PEDOT:PSS is a commercially available polymer in the form of an emulsion, it can be easily used as a binder in the cathode coating fabrication under any coating fabrication technique, such as via doctor blade or spraying. For this reason, it has been selected to be investigated as a cathode binder for Li–S batteries in this study.

The scope of this study is to investigate the role of PEDOT:PSS as an energy extender in Li–S batteries with cathodes of hollow porous carbon particles impregnated with sulfur and processed to a coating form using a PEDOT:PSS binder. An existing multipore continuum model developed by our group^7,18,19^ will be extended to a novel model to include the effect of the PEDOT:PSS binder considering Li^+^ ion intercalation and surface redox as in our single particle model (SPM) algorithm^3^ and the adsorption energy of PEDOT:PSS with respect to sulfur and sulfides. Molecular modeling and simulations will be conducted to determine the adsorption energies and other parameters to populate the continuum model. Finally, simulations based on the novel continuum model were employed to elucidate the effects of the PEDOT:PSS binder on a galvanostatic discharge–charge cycle of the Li–S battery cell. A parallel experimental study will link and relate experimental data for Li–S cells, a supercapacitor with electrodes containing the PEDOT:PSS binder and the same electrolyte as the Li–S cell, and simulation predictions.

## 2 Experimental Methods

### 2.1 Materials

The main cathode host in the Li–S battery cells and the basis and the main component of the supercapacitor cells was Ketjenblack EC-600JD (Lion Corporation, Japan), denoted as KB in this paper, which was supplied as a powder of hollow porous carbon particles of diameter about 30 nm, with 80% hollow core, a total specific volume of 2.9 cm^3^ g^–1^, a specific surface area SSA_BET_ = 1415 m^2^ g^–1^, and a pore size distribution (PSD) given in ref (18). Sulfur was supplied as a powder (Sigma Aldridge, U.K.). PEDOT:PSS Clevios PH 1000 (Heraeus, Germany) was supplied as a 1–1.3 wt % aqueous emulsion. The current collector for the supercapacitor electrodes and the sulfur cathode was carbon-coated aluminum foil (MTI). The electrolyte was 1 M LiTFSI (lithium bis(trifluoromethanesulfonyl)imide) in DOL/DME (1,3-dioxolane/dimethoxyethane) 1:1 v/v, with also 0.8 M LiNO_3_. The separator was one layer of Celgard 2400 (Celgard) of 25 μm thickness with a porosity of 41% and a mean slit pore width of 44 nm.^36^ The same electrolyte and separator were used in both the supercapacitor cells and Li–S cells. A lithium foil anode (Sigma-Aldrich, U.K.) was used in the Li–S battery cell.

### 2.2 Fabrication

Supercapacitor electrodes of 90 wt % KB and 10 wt % PEDOT:PSS were fabricated via the doctor blade technique at a gap of 250 μm. With regard to the sulfur cathode, the KB powder was lightly ground and mixed with sulfur powder at the required ratio in a pestle and mortar for half an hour. The mixture was placed in a tray sealed/covered with aluminum foil and heated in an oven for 2 h at 155 °C. The resulting mixture was ground in a pestle and mortar for 30 min and dispersed in the PEDOT:PSS aqueous emulsion. The slurry was dispersed in an ultrasound bath for 15 min and left under magnetic stirring, slowly evaporating the water until a paste was formed. The cathode coating was fabricated via a doctor blade technique at a gap of 250 μm. The final cathode contained 45 wt % sulfur, 45 wt % KB, and 10 wt % PEDOT:PSS.

Supercapacitor cells and Li–S cells were fabricated in the form of circular cells of 19.2 mm electrode diameter and 25 mm separator diameter. A two-part cell case was employed, as presented in ref (37) (SI file). The supercapacitor cells were symmetric and fully flooded with the electrolyte. As their electrodes consisted of high surface area KB material and the pseudocapacitive PEDOT:PSS binder, these supercapacitor cells are considered hybrid pseudocapacitive-electrical double-layer capacitor (EDLC) cells. The Li–S cells had an electrolyte at an electrolyte-to-sulfur ratio E/S = 11 μL g_S_^–1^, which, combined with the microstructural data of the cathode and separator, was estimated to yield just a fully saturated cathode and a separator for the model and computer simulations.^19^

### 2.3 Testing

The KB powder was characterized by using a Thermo Fisher Scientific Talos F200i scanning transmission electron microscope (STEM). A 200 keV beam was used in STEM mode at “spotsize” 5, with a selected condenser2 aperture size of 70 μm. The resulting screen current was ∼300 pA. Energy-dispersive X-ray spectroscopy (EDX) maps were acquired by using a Bruker X-Flash detector. The cathode coating was subjected to FIB (focused ion beam) followed by SEM/EDX employing an HR-SEM JEOL-7100 F.

The supercapacitor cells and Li–S cells were subjected to electrochemical testing. This included electrical impedance spectroscopy (EIS) tests in the frequency range of 10 mHz to 1 MHz, also galvanostatic charge–discharge (GCD) tests for the supercapacitor cells and galvanostatic discharge–charge (GDC) tests for the Li–S cells. The GCD and GDC tests were conducted at different current densities, with the current densities translated to the C-rate with respect to the mass of sulfur for the Li–S cells.

## 3 Multiscale Modeling Methods

### 3.1 Molecular Modeling and Simulations

A PEDOT:PSS structure of five repeating units was constructed and optimized geometrically in Materials Studio 6.1 (Accelrys). The PEDOT:PSS structure, as well as structures of Li_2_S, Li_2_S_2_, Li_2_S_4_, Li_2_S_6_, and Li_2_S_8_ were optimized geometrically in *ab initio* simulations in CASTEP v19.1.1 (CASTEP.org, U.K.). Pairs of the PEDOT:PSS structure and each of these lithium sulfides were inputted in the Blends Module of Materials Studio,^38^ which was run in “superfine” simulations to determine the coordination number, n_C_, of each of the above Li_2_S_*x*_ molecules and also Li and determine the first set of optimized structures of PEDOT:PSS/Li_2_S_*x*_. These optimized structures were used as an initial guess in *ab initio* spin-polarized density functional theory (DFT) simulations using CASTEP v19.1.1 in which the generalized gradient approximation (GGA) with the Perdew–Burke–Ernzerhof (PBE) functional was employed and Brillouin zone integrations were conducted with a tight k-point separation of 0.04 Å^–1^^39^ A plane-wave cutoff energy of 500 eV and tight convergence criteria of 10^–5^ eV for energy and 0.5 × 10^–4^ eV Å^–1^ for force tolerance were employed.^22^ The van der Waals dispersion corrections were taken into account, as described in Grimme’s empirical method.^40^ The average adsorption energy between any Li_2_S_*x*_ and PEDOT:PSS, *E*_ads_ (in kJ per mol of Li_2_S_*x*_), was calculated from the difference between the minimum energy of the combined structure, *E*_PEDOT:PSS/Li_2_S*_x_*_, and the individual minimum energies of each optimized structure, *E*_PEDOT:PSS_ and *E*_Li_2_S*_x_*_, also taking into account the coordination number of the corresponding Li_2_S_*x*_ surrounding the PEDOT:PSS structure, *n*_C,Li_2_S*_x_*_, according to the relation
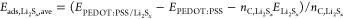
1

### 3.2 Continuum Model and Simulations

A continuum, two-phase model is employed in this study with phase volume averaged equations for the main variables, such as current and voltage, for both the supercapacitor cell and the Li–S battery cell. This model is based on the multipore models developed by our group for EDLCs^38,41,42^ and Li–S batteries.^7,18,19^ It is further extended in this study to incorporate the role of the PEDOT:PSS binder.

#### 3.2.1 Hybrid Pseudocapacitor-EDLC Model

**Figure 1:**
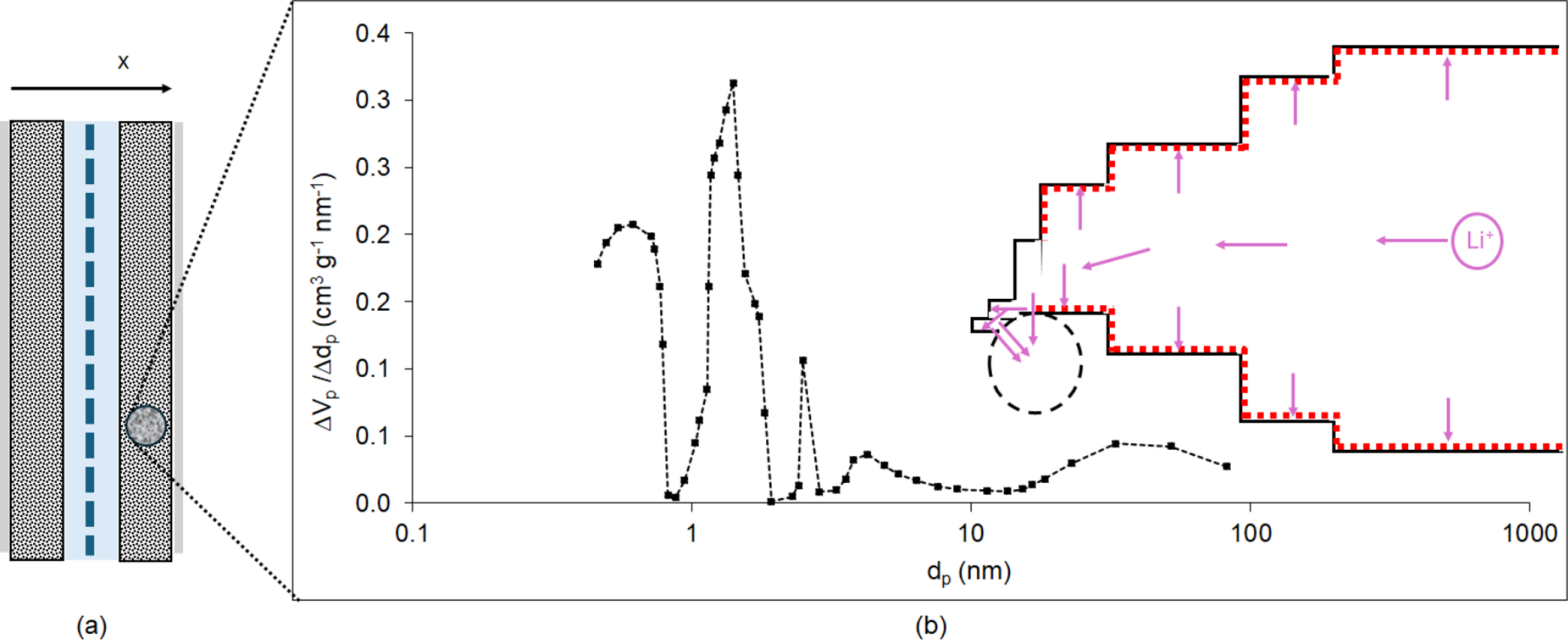
Concept of the multipore continuum model for cycling of the hybrid pseudocapacitor-EDLC. (a) 1d-continuum model in the *x*-through thickness direction of the cell consisting of outer current collector foils, electrodes, separator, and liquid electrolytes. (b) The electrode microstructure detail of an *x*-location in the cell, represented by a discretized PSD in which the ions are transported along the pore size hierarchy until the pore size of the core of the hollow particle exchanges transported ions with all smaller pores, which are part of the porous wall of the hollow particles. Furthermore, the walls of pores greater than 30 nm (particle size) are coated with a nanolayer of the PEDOT:PSS binder (red lining in pore diagram).

The diagram in Figure 1 presents the concept of the continuum model in this study. It is a multipore model that involves ion transport following a pore line model along the pore size hierarchy,^38,41,42^ with a discretized PSD inputted in the simulations of this study presented in Figure 1b. Given the nature of the hollow porous particles, the pore size of 22.8 nm is inputted as the last pore size, which is fed from or feeds ions to all of the smaller pores in charge and discharge, respectively, which are part of the porous particle wall. It is also considered that the PEDOT:PSS binder coats each particle and particle aggregates and agglomerates; hence, as shown in Figure 1b, it lines the walls of all pores greater than 30 nm, which is considered to be the size of the interparticle gaps for particles of average size of 32.9 nm.

The multipore continuum model for the hybrid pseudocapacitor-EDLC device is based on the ion transport equation for species s (electrolyte ions Li^+^ or TFSI^–^) in pore size p for pores smaller than the interparticle gap (30 nm), as proposed by^38,41,42^
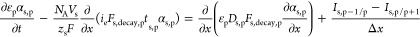
2This is solved for the volume fraction of species s in pore p, α_s,p_, based on the electrolyte current density, *i*_e_, decay factor *F*_S,Decay_, and interpore fluxes of species s from pore p-1 to pore p, *I*_s,p-1/p_, as described in refs (38,41,42). Additionally, *x* is the direction through the cell thickness, *t* is time, *N*_A_ is the Avogadro number, *V*_s_ is the molecular volume of species s, *z*_s_ is the charge of species s, and *F* is the Faraday constant.

The following novel features have been added to our multipore continuum EDLC model to extend it for hybrid pseudocapacitor-EDLC devices.

For pores p greater than or equal to the interparticle gap (>30 nm), the presence of the PEDOT:PSS binder coating the pore walls leads to the addition of two extra terms in eq 2 for the Li^+^ ion transport: transverse diffusion through the PEDOT:PSS nanolayer coating and surface redox reaction of Li^+^ ions with PEDOT:PSS
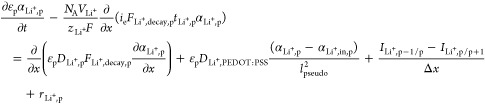
3The Li^+^ ion volume fraction varies through the *y*-direction, i.e., the direction through the thickness of the PEDOT:PSS nanolayer coating the pore wall, from α_Li^+^,p_ at the PEDOT:PSS surface in contact with the liquid electrolyte to α_Li^+^,in,p_ at the PEDOT:PSS surface in contact with the solid surface of the KB particle or aggregate. α_Li^+^,in,p_ is determined from the solution of the following diffusion equation through the PEDOT:PSS nanolayer, which is in cylindrical coordinates in relation to the walls of the meso- and macropores (>30 nm) that are considered cylindrical in this study
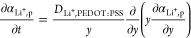
4*D*_s,p_ is the diffusion coefficient of species s in the liquid electrolyte in pore p of size *d*_p_, given by a modified Stokes–Einstein equation.^19,38,41,42^ With regards to the diffusion coefficient of Li^+^ ions through the PEDOT:PSS wall layer in pores p greater than 30 nm (which is the size of KB particles and assumed interparticle gaps), this was taken as *D*_Li^+^,PEDOT:PSS,intercalation_ = 1 × 10^–20^ cm^2^ s^–1^ during intercalation and *D*_Li^+^,PEDOT:PSS,deintercalation_ = 1 × 10^–21^ cm^2^ s^–1^ during deintercalation. These values of Li^+^ ion diffusion coefficient were determined using the Rendles–Shevchik eq 3 and CV data^31^ of the supercapacitor with the Li-ion electrolyte and electrodes with the 10 wt % PEDOT:PSS binder, which was fabricated via the doctor blade technique.

For pores p greater than 30 nm, lined with PEDOT:PSS, the following surface redox reaction is supposed to take place as found for doctor blade-fabricated electrode coatings with the 10 wt % PEDOT:PSS binder and the Li-ion electrolyte^31^
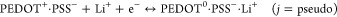
Hence, for these pores p greater than 30 nm, the rate of this electrochemical reaction, *r*_s,p_, is included in eq 2, with s referring only to Li^+^ ions and *r*_s,p_ is given by
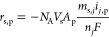
5where *n*_*j*_ is the number of electrons transferred in reaction *j* (*j* = pseudo in this case and *n_j_* = 1 for the surface redox of Li^+^ and PEDOT:PSS), *m*_s,*j*_ is the stoichiometric coefficient of species *s* in electrochemical reaction *j*, and *A*_p_ is the specific area of the porous cathode in pore p. TFSI^–^ ions do not participate in any reactions in the model, hence, *r*_TFSI-,p_ = 0. The current density due to the pseudocapacitor redox reaction in pore p, *i*_pseudo,p_, is given by the Butler–Volmer equation
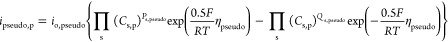
6where *C*_s,p_ is the concentration of species s in pore p given by the following relation
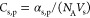
7*P*_s,*j*_ = *m*_s,*j*_ for anodic species and *Q*_s,*j*_ = −*m*_s,*j*_ for cathodic species, *i*_o,pseudo_ is the exchange current density of electrochemical reaction *j* = pseudo, and η_pseudo_ is the overpotential for reaction *j* = pseudo given by the following equation
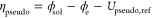
8where ϕ_sol_ and ϕ_e_ are the potential of the solid and liquid phase, respectively, and *U*_pseudo,ref_ is the open-circuit potential (OCP) for reaction *j* = pseudo. The reaction kinetic parameters *i*_o,pseudo,ref_ and *U*_pseudo,ref_ were determined in this study by fitting the simulation predictions with experimental GCD data for a hybrid pseudocapacitor-EDLC cell.

Input data for the electrolyte ion dimensions in solvated and desolvated form, desolvation energies, and coordination numbers were obtained from ref (43). Data for the physical properties of the electrolyte, such as viscosity, ionic conductivity, and dielectric constant were obtained from ref (44).

#### 3.2.2 Li–S Battery Model

**Figure 2:**
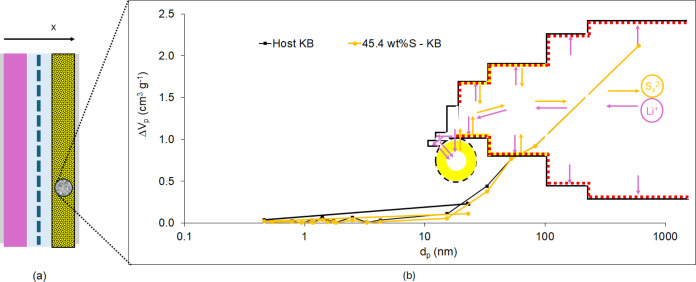
Concept of the multipore continuum model for the cycling of the Li–S battery cell. (a) 1d-continuum model in the *x*-through thickness direction of the cell consisting of outer current collector foils, electrodes (Li anode and S-KB cathode), a separator, and a liquid electrolyte; (b) 45 wt % S-KB microstructure detail of an *x*-location in the cell, represented by a discretized PSD in which the ions are transported along the pore size hierarchy, until the pore size of the core of the hollow particle exchanges transported ions with all smaller pores which are part of the porous wall of the hollow particles. Furthermore, the walls of pores greater than 30 nm (particle size) are coated with a nanolayer of the PEDOT:PSS binder (red lining in pore diagram). Transport of Li^+^ ions toward the cathode and S_*x*_^2–^ ions away from the cathode is illustrated during discharge.

Figure 2 presents the continuum multipore model concept for the Li–S battery cell of this study. The same multipore continuum model is applied to the Li–S battery cell as to the hybrid EDLC-pseudocapacitor, as described in Section 3.2.1. The model is represented by the main species s transport equation
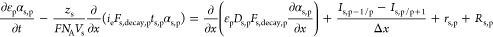
9eq 9 applies to the following s species: electrolyte ion species, Li^+^ and TFSI^–^, sulfur allotropes S_8_ and S_4_ depending on accommodating pore size, and sulfides S_8_^2–^, S_6_^2–^, S_4_^2–^, S_2_^2–^, and S^2–^. It includes the same terms as eq 2, with the additional terms of the rate of species dissolution or precipitation, *R*_s,p_, given in refs (7,18,19) and dissolution data from refs (4,45) and the rate of species production or consumption, *r*_s,p_, including the Li–S battery reactions as presented in refs (7,18,19) and the additional surface redox reaction of Li^+^ ions with PEDOT:PSS (*j* = pseudo) as presented in Section 3.2.1. Another novel feature added in this study includes embedding the effect of species adsorption energy with the PEDOT:PSS lining of the pore walls, *E*_ads,Li_2_S_x__, in the species decay factor, *F*_s,Decay,p_, with s = Li_2_S_*x*_, as follows
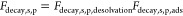
10This means that the decay factor, *F*_decay,s,p_, which is a multiplier in the drift current migration and the diffusion term in the transport eq 9, is the product of the decay factor associated with the desolvation of species when moving to pores smaller than the solvated species^7,18,19^ and the decay factor associated with the adsorption energy of Li_2_S_*x*_ with the PEDOT:PSS lining of the pore walls, *F*_decay,s,p,ads_, when s = Li_2_S_*x*_, given by
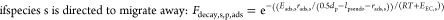
11
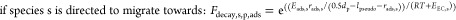
12*E*_ads,s_ is given from the predictions of DFT simulations presented in Figure 6, *r*_ads,s_ is the distance of s (Li_2_S_*x*_) species from PEDOT:PSS at its minimum energy state extracted from the DFT optimized structures in Figure 5, where it is assumed that *E*_ads,s_ falls inversely with the distance from the optimized minimum energy position of Li_2_S_*x*_ with respect to PEDOT:PSS (Figure 5), depending on pore size *d*_p_ and given by 0.5*d*_p_ – *l*_speudo_ – *r*_ads,s_. *E*_EC,s_ is the electrochemical energy associated with the electric field on species s.

## 4 Results

### 4.1 Materials Characterization

**Figure 3:**
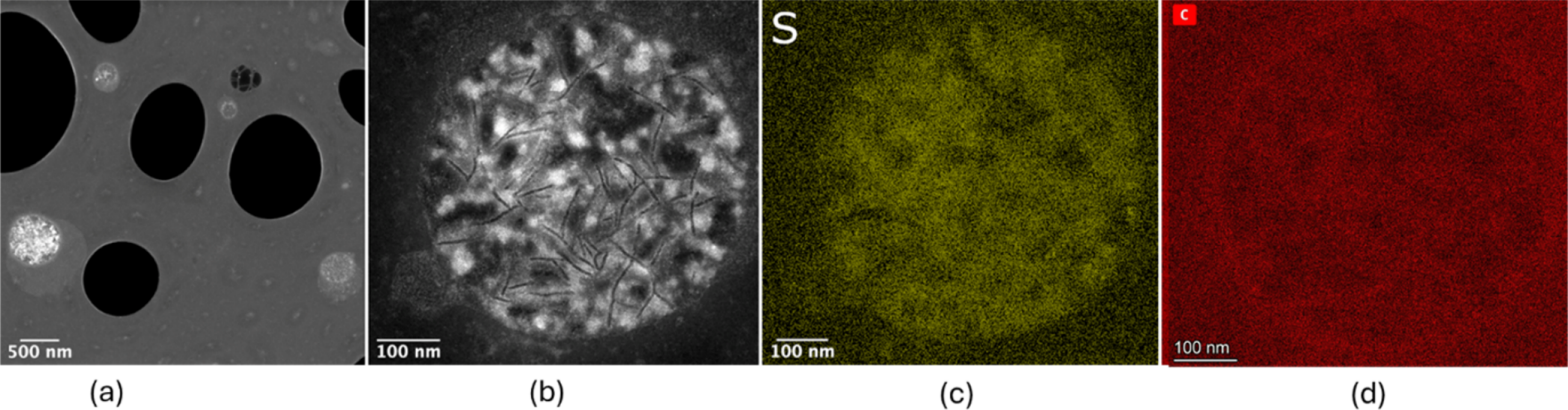
STEM/EDX images of sulfur-impregnated KB powder (S:KB at 50:50 g/g): (a) the STEM image of particle agglomerates on the support grid; (b) the HAADF-STEM image of a particle agglomerate; (c) the S-EDX map of image (b); and (d) the C-EDX map of image (b).

STEM characterization of the ground sulfur-infiltrated KB mixture yielded spherical agglomerates of S-KB particles (S/KB 50:50 g/g) of size in the range 200–550 nm, as shown in Figure 3a. Figure 3b shows a magnified high-angle annular dark-field (HAADF) STEM image that clearly depicts the sulfur-infiltrated KB particles with brighter spots indicating the presence of sulfur within the KB particles. This is confirmed by the EDX S-element map in Figure 3c coinciding with the C-element map in Figure 3d, where the S-map pattern parts are somehow slimmer than or at least coincide with the C-map pattern, indicating that sulfur has infiltrated the core of the KB particles.

**Figure 4:**
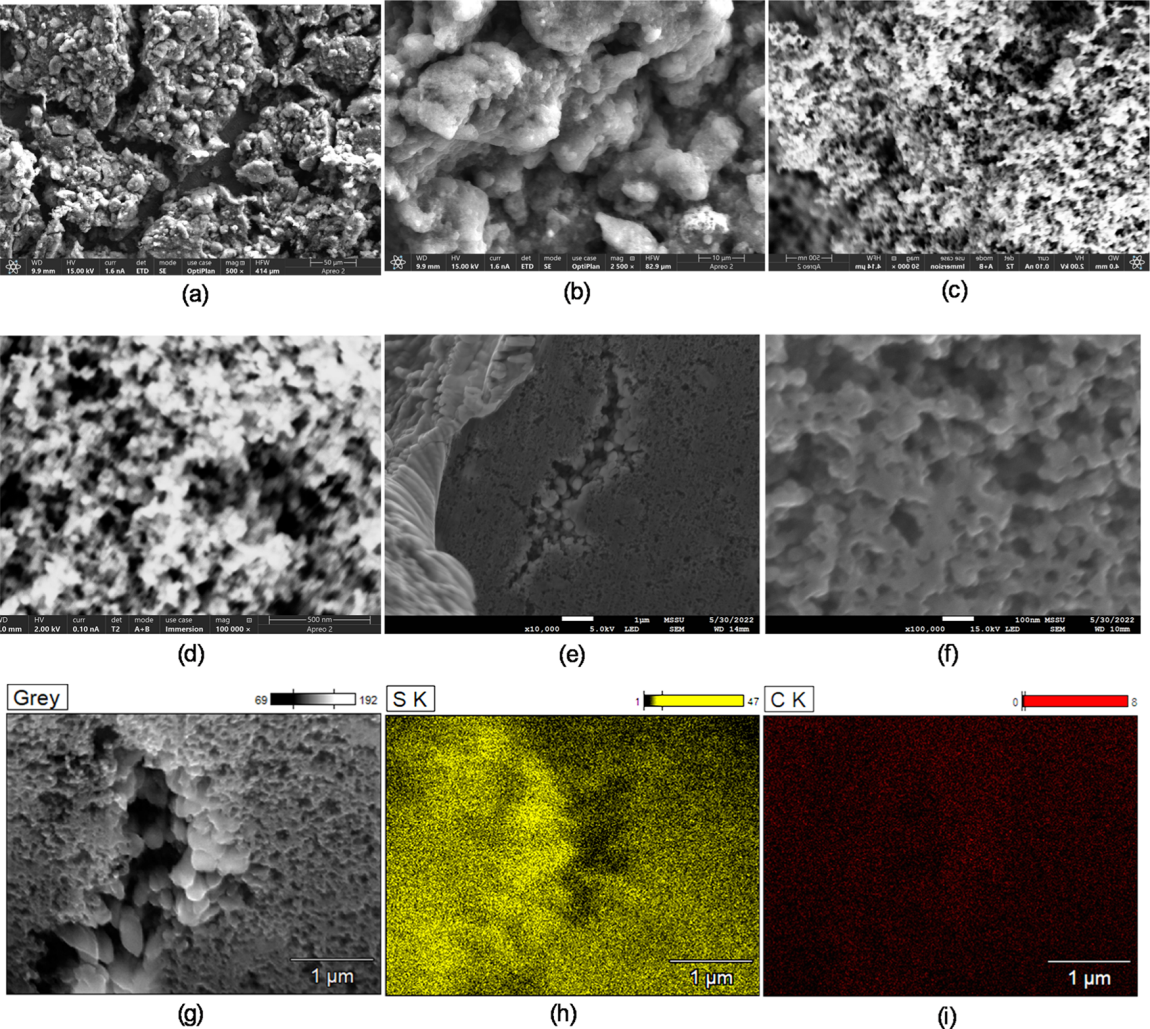
SEM/EDX images of 45 wt % S-KB coating: (a–d) SEM images of different magnifications; (e–g) FIB/SEM images, scale: 1 μm, 100 nm, 1 μm, respectively; (h) the S-EDX map of image (g); and (i) the C-EDX map of image (g).

Figure 4 presents images from the characterization of the cathode coating with 45 wt % S. Figure 4a depicts cracks of about 30 μm width in the coating, defining coating domains of 200–300 μm. Each such domain seems to contain aggregates with interaggregate gaps from 10 to 5 μm down to 1 μm and further down to 200, 100, and 50 nm, as depicted in Figure 4b–d,f. Figure 4e presents an SEM image of the 45 wt % S-KB cathode coating with a focus on the region milled by FIB, depicting a small coating crack in the middle with dislodged S-KB agglomerates. Figure 4f focuses on the part of the FIB-milled region rich with a network of S-KB particles, with the sectioned particles exhibiting a flat section surface, implying good impregnation of the core of the hollow KB particles with sulfur. Figure 4g–i presents an SEM image of the FIB-milled region around the crack and the corresponding S- and C-element EDX maps, respectively, demonstrating the prominent presence of the S-element in the particle core and surface as well as surrounding the particle surface. This is in contrast to the S- and C-EDX maps in Figure 3, where S seemed to be confined in the KB particles. The differences in the S-element distribution between Figures 3 and 4 might be attributed to the fact that the cathode coating in Figure 4 also contains elemental S from PEDOT:PSS that seems to wrap the external surface of individual KB particles. Taking into account the specific surface area of pores equal to or greater than 32.9 nm from the PSD of the coating corresponding to interparticle gaps and assuming that the full amount of the PEDOT:PSS binder has been distributed homogeneously around the external surface of the S-KB particles, a PEDOT:PSS nanolayer of average thickness *l*_pseudo_ = 3.3 nm is estimated. Figure 4h in relation to Figure 4g leads to estimates of a thicker PEDOT:PSS binder layer up to about 20 nm.

### 4.2 Molecular Modeling and Simulation Results

**Figure 5:**
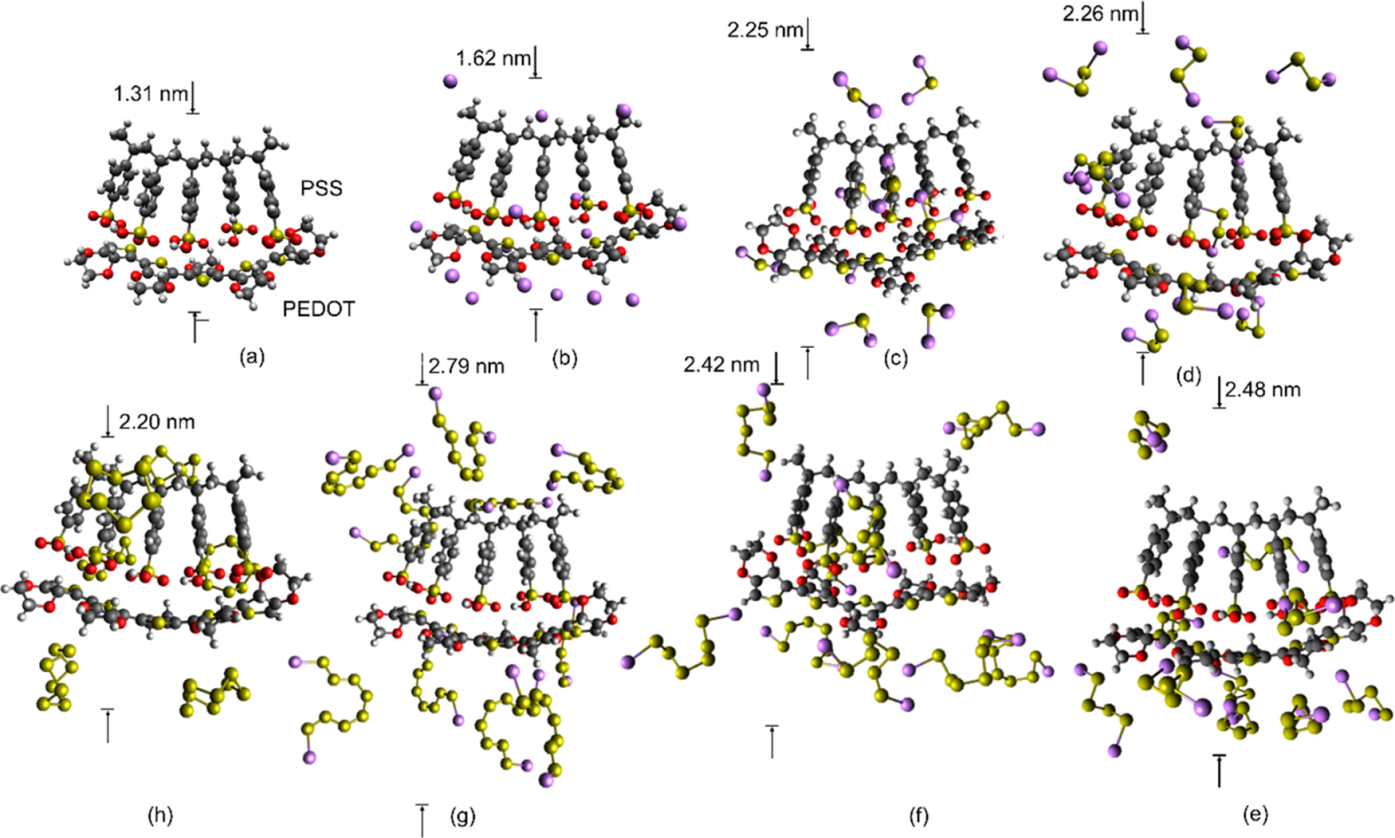
Geometrically optimized molecular structures: (a) PEDOT:PSS, (b) PEDOT:PSS with 16 coordinated Li atoms, (c) PEDOT:PSS with 13 coordinated Li_2_S molecules, (d) PEDOT:PSS with 12 coordinated Li_2_S_2_ molecules, (e) PEDOT:PSS with 11 coordinated Li_2_S_4_ molecules, (f) PEDOT:PSS with 10 coordinated Li_2_S_6_ molecules, (g) PEDOT:PSS with 10 coordinated Li_2_S_8_ molecules, and (h) PEDOT:PSS with 10 coordinated S_8_ molecules.

Figure 5 presents the optimized structures of a PEDOT:PSS segment with five repeating units and combined structures of this PEDOT:PSS segment with the full number of coordinated molecules of Li, Li_2_S, Li_2_S_2_, Li_2_S_4_, Li_2_S_6_, and Li_2_S_8_. The electrostatic interactions between the sulfonate anions of PSS and PEDOT that carry and conduct positive charges create lamellar PEDOT:PSS structures of alternating PEDOT and PSS layers or PEDOT-rich regions and PSS-rich regions.^46,47^ Such interactions have formed the tight structure in Figure 5a with a transverse dimension of 1.3 nm.

**Figure 6:**
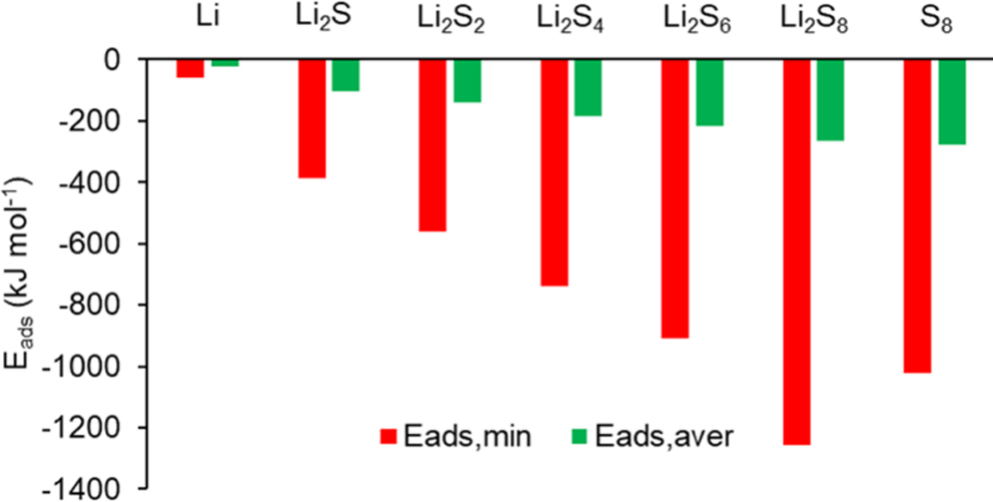
Adsorption energies of Li and Li_2_S_*x*_ with regards to PEDOT:PSS: minimum and average *E*_ads_ values.

The coordination with Li atoms in Figure 5b is favored by the Li···O and Li···S secondary bonds. Coordination with Li_2_S_*x*_ molecules in Figure 5c–g is favored by Li···O and Li···S secondary bonds with regards to the Li part of Li_2_S_*x*_ and by electrostatic interactions between the positive carrier PEDOT and the electronegative S_*x*_ part of Li_2_S_*x*_. Figure 6 presents the adsorption energies predicted by the DFT simulations, where it can be seen that the adsorption energy increases with the length of the polysulfide. Figure 6 presents both the minimum *E*_ads_ values and the average *E*_ads_ values averaged over all coordinated molecules of the adsorbed species by PEDOT:PSS, as shown in eq 1. The *E*_ads,ave_ values were used as input data in the continuum model simulations.

### 4.3 Supercapacitor Test and Simulation Data

**Figure 7:**
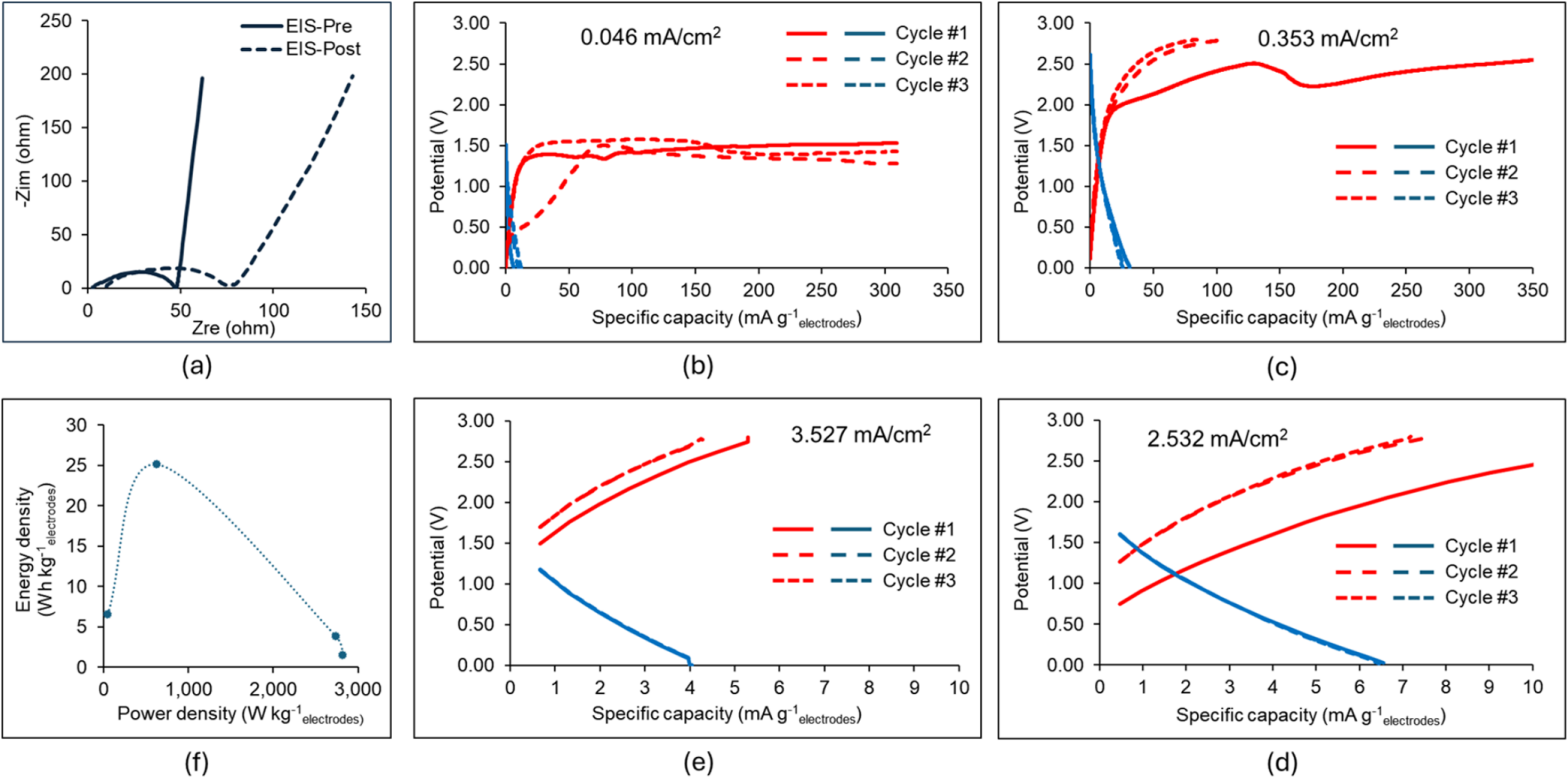
Results of the electrochemical testing of symmetric supercapacitor cell with electrodes based on 90 wt % KB-10 wt % PEDOT:PSS coating and electrolyte 1 M LiTFSI in DOL:DME 1:1 v/v. (a) Nyquist plots from EIS data of as-fabricated cell (EIS-Pre) and post-mortem after all GCD cycles (EIS-Post); (b–e) data of galvanostatic charge–discharge tests at different current densities (red: charge curves, blue: discharge curves); and (f) the Ragone plot based on the galvanostatic discharge test data.

Figure 7 presents the experimental data from the EIS and GCD testing of the hybrid pseudocapacitor-EDLC cells. The GCD data demonstrate a strong pseudocapacitive behavior during charge at low current densities of 0.046 and 0.353 mA cm^–2^ that becomes negligible at a high current density of 3.5 mA cm^–2^. However, this pseudocapacitive behavior is not reversed in discharge in the tested potential difference down to a minimum cell potential of 0 V at the end of discharge. The Nyquist plot in Figure 7a exhibits an increased equivalent in series resistance (ESR) of the supercapacitor cell after cycling, from 47.6 ohm for the as-fabricated cell (EIS-Pre) to 77.3 ohm after the GCD cycling (EIS-Post). This rise in ESR is attributed to the higher resistance of the nonreversed PEDOT^0^·PSS^–^·Li^+^ to the original PEDOT^+^·PSS^–^ in the binder of the as-fabricated cell.

**Figure 8:**
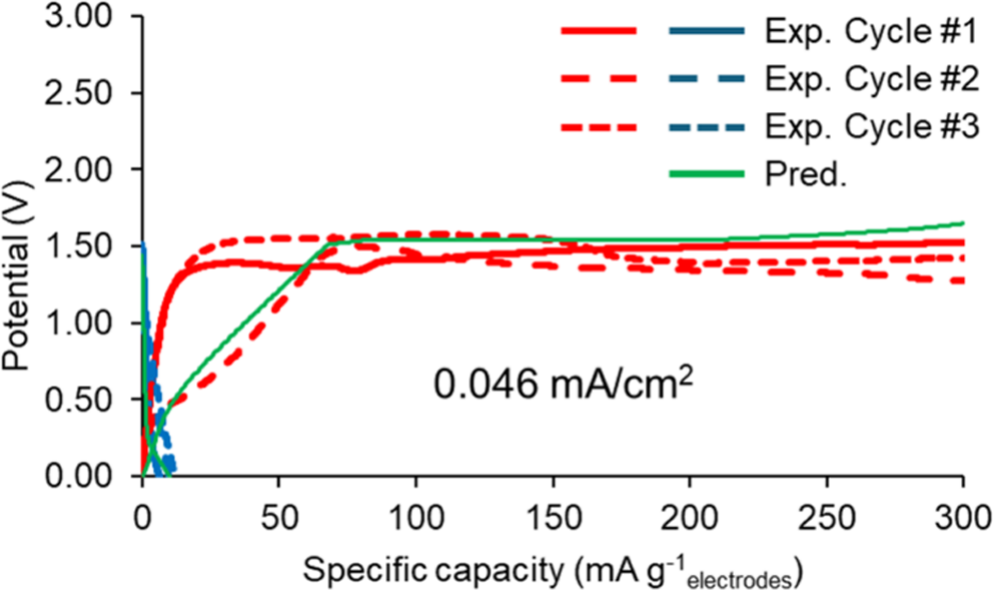
Predictions from continuum model simulations against experimental data of the GCD tests of the symmetric supercapacitor cell with electrodes based on 90 wt % KB-10 wt % PEDOT:PSS coating and electrolyte 1 M LiTFSI in DOL:DME 1:1 v/v at a current density of 0.046 mA cm^–2^.

Simulations of the KB-based supercapacitor with the PEDOT:PSS binder revealed that there is negligible intercalation and diffusion of Li^+^ ions in the anode, even when the lowest possible value of the PEDOT:PSS wall nanolayer *l*_pseudo_ = 3.3 nm is considered. Hence, only the surface redox reaction *j* = pseudo was incorporated in the continuum multipore model for the hybrid pseudocapacitor-EDLC. After a few trial-and-error simulation runs, the following parameter values were selected for the surface redox *j* = pseudo
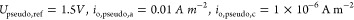
The selected value of *U*_pseudo,ref_ is close to the plateau voltage of the charge curves at low current density in Figure 7b and within the range of 1.4–1.6 V for the Li^+^ ion intercalation in the CV plots in our past study.^31^ Guided by the experimental data in Figure 7 that indicated an almost irreversible surface redox reaction of Li^+^ ions with PEDOT:PSS, different exchange current density values were set for the anodic and cathodic redox reactions, *i*_o,pseudo,a_ and *i*_o,pseudo,c_, respectively. Figure 8 presents the GCD predictions from continuum model simulations using these surface redox reaction parameter values and compares them to the experimental GCD data. The predictions exhibit good agreement with the experimental data of the third-cycle charge and second-cycle discharge. The simulation was conducted at a low current density at which surface redox is present in the experimental data. These surface redox parameter values were employed subsequently in the continuum model simulations for the Li–S battery cell in which PEDOT:PSS is present at 10 wt % as a binder in the cathode.

### 4.4 Li–S Battery Cell Test and Simulation Data

**Figure 9:**
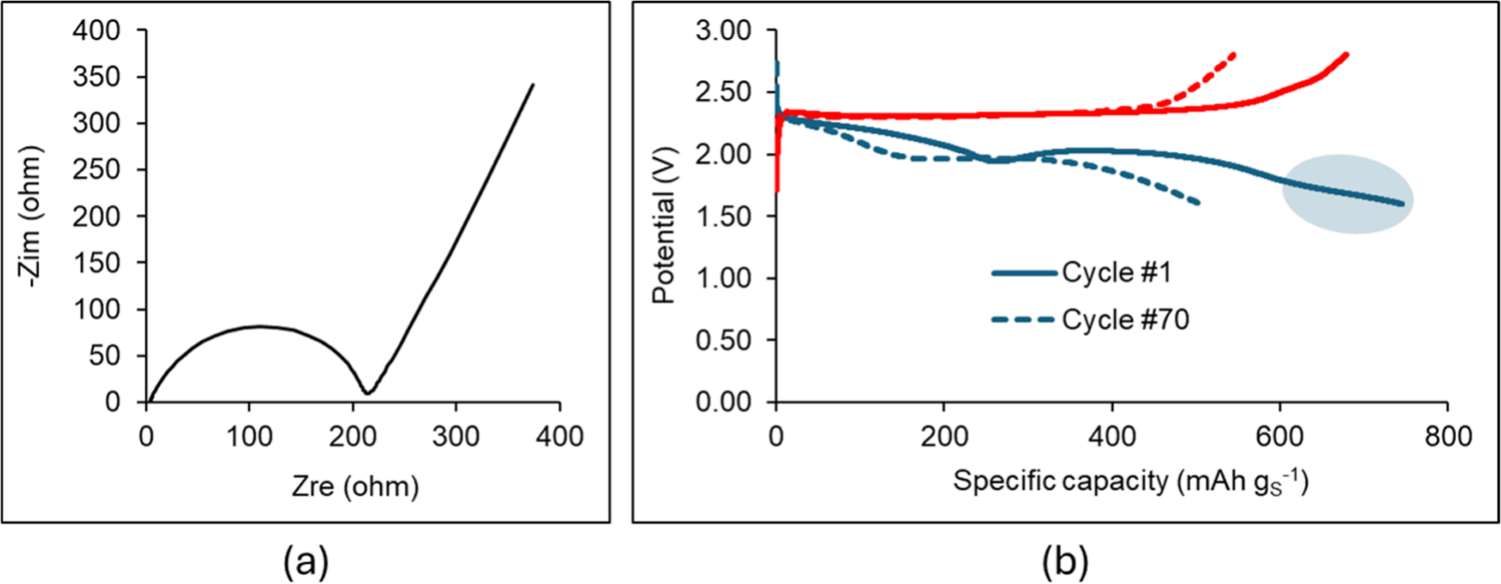
Results of the electrochemical testing of Li–S cell with the cathode of 45 wt % S-45 wt % KB-10 wt % PEDOT:PSS coating and electrolyte 1 M LiTFSI in DOL:DME 1:1 v/v. (a) The Nyquist plot from EIS data of the as-fabricated cell (EIS-Pre) and (b) data of galvanostatic discharge–charge tests at 0.05 C.

Figure 9 presents the experimental data from electrochemical testing of the Li–S battery cell. Figure 9a displays the Nyquist plot of the as-fabricated cell (pre-GDC cycling) in which an ESR of 216 ohm is determined. Compared with the ESR of 47.6 ohm of the as-fabricated corresponding supercapacitor in Figure 7a, the additional resistance of 168 ohm is mainly attributed to the 45 wt % sulfur content of the cathode, with sulfur being an electrical insulator. Figure 9b shows that the first discharge at 0.05 C yields a specific capacity of 744 mAh g_S_^–1^, which is reduced to 678 mAh g_S_^–1^ with the first charge. The discharge capacity is reduced by 32.5% after 70 GDC cycles, although the charge capacity is 8% higher than the discharge capacity in the 70th GDC cycle, indicating a possible effect of “shuttling” polysulfides during the charge.

**Figure 10:**
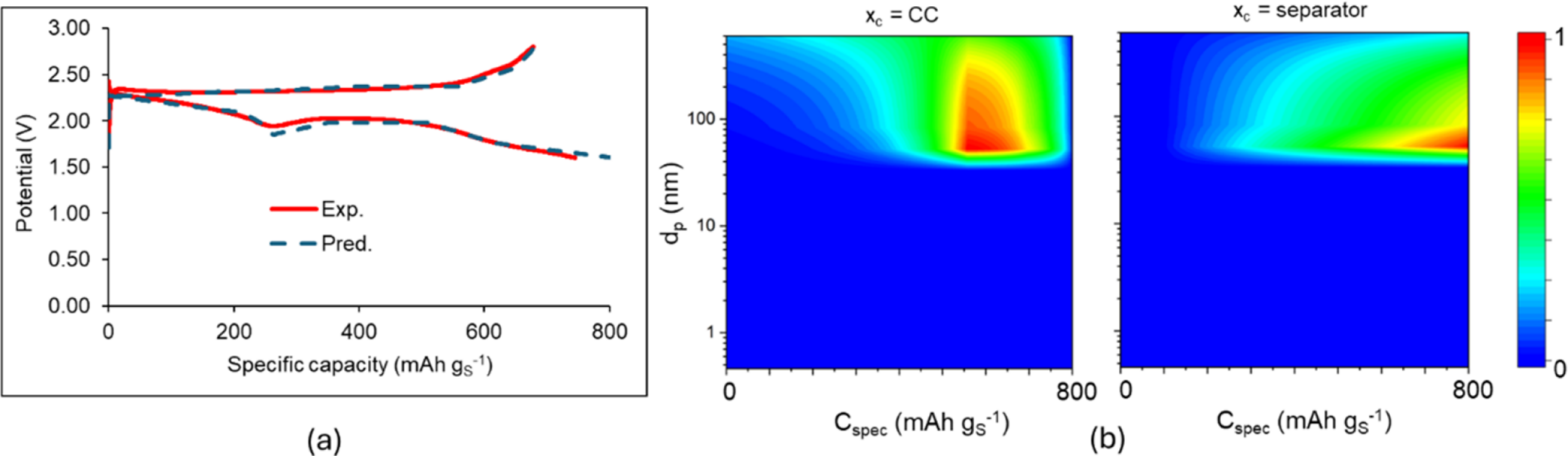
Predictions from continuum model simulations against experimental data of the first GDC cycle of the Li–S cell with a cathode of 45 wt % S-45 wt % KB-10 wt % PEDOT:PSS coating and electrolyte 1 M LiTFSI in DOL:DME 1:1 v/v at 0.05 C. (a) GDC curves for the first GDC cycle: predictions versus experimental data; (b) colored contour maps of the fraction PEDOT^0^·PSS^–^·Li^+^/PEDOT^+^·PSS^–^ as a function of the specific capacity during the first discharge and the cathode pore size at two different positions in the cathode: *x*_c_ = CC by the cathode current collector and *x*_c_ = separator: cathode surface by the separator.

Regarding the Li–S model and simulations, the transport of Li^+^ ions to the cathode of the Li–S cell and subsequent surface redox with the PEDOT:PSS binder in the cathode during discharge are equivalent to the same processes taking place in the anode of the hybrid pseudocapacitor-EDLC during its charge phase. *U*_pseudo,ref_ = 1.54 V against Li/Li^+^ was inputted in the simulations of the Li–S cell, which is equivalent to *U*_pseudo,ref_ = 1.5 V optimized for the supercapacitor cell against the counter electrode of the supercapacitor. Figure 10a presents the predictions from continuum model simulations of the first GDC cycle, which show very good agreement of the predicted GDC curves with the experimental data at 0.05 C. Figure 10b shows the predicted contour plots of the fraction of the surface lithiated PEDOT:PSS during the first discharge at two different positions in the cathode: by the current collector and by the separator. As expected, the surface redox process on PEDOT:PSS is stronger by the current collector than by the separator due to the higher potential difference versus Li/Li^+^ at the cathode current collector than at the separator. Both plots exhibit a prediction of peaking of the surface redox reaction after 600 mAh g_S_^–1^ at discharge, which is consistent with the last part of the slope change in the discharge curve in the experimental discharge curve in Figure 9b.

**Figure 11:**
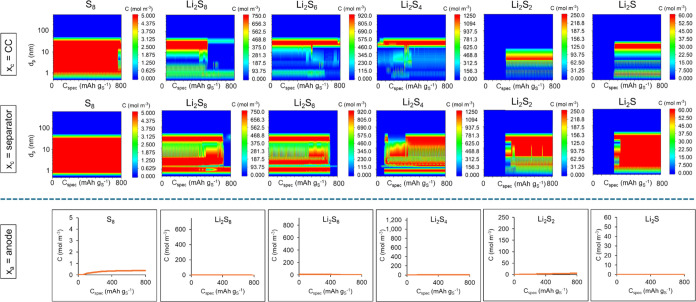
Predicted concentrations of the dissolved sulfur and sulfides in the electrolyte solution as a function of specific capacity during the first discharge of the Li–S battery cell with a cathode of 45 wt % S-45 wt % KB-10 wt % PEDOT:PSS coating: contour plots in the cathode as a function of pore size for two different locations (by the current collector and by the separator) and concentration profiles at the anode. The maximum concentration limit in each plot is set to the saturation concentration of that species in the liquid electrolyte.

Figure 11 presents the predicted concentrations of sulfur and sulfides as contour plots for different pore sizes at two positions in the cathode and as profiles in the anode, both contour plots and profiles being a function of the specific capacity. It can be seen that sulfur is being dissolved in the cathode until the end of discharge due to remaining undissolved sulfur after 800 mAh g_S_^–1^, which is the main reason why the Li–S cell does not reach the full theoretical capacity of 1672 mAh g_S_^–1^ in the first discharge. The continuous dissolution of sulfur during the whole discharge generates Li_2_S_8_ and Li_2_S_6_ in various pore sizes, all the way to high capacities.

It seems that there is some migration of the soluble sulfides through the cathode from the current collector side to the cathode surface by the separator, which causes the concentration on the cathode surface to exceed the saturation concentration and a layer of sulfide deposits to be formed on the cathode surface. A small degree of migration of the soluble sulfur and a very small degree of migration of soluble sulfides continue to the anode, as displayed in the predicted concentration profiles at the anode in Figure 11. This might create higher sulfide concentrations at the anode in subsequent cycles and continue as shuttling of the soluble sulfides during charge in subsequent cycles, explaining the longer charge curve in the 50th GDC cycle in the experimental data of Figure 9.

**Figure 12:**
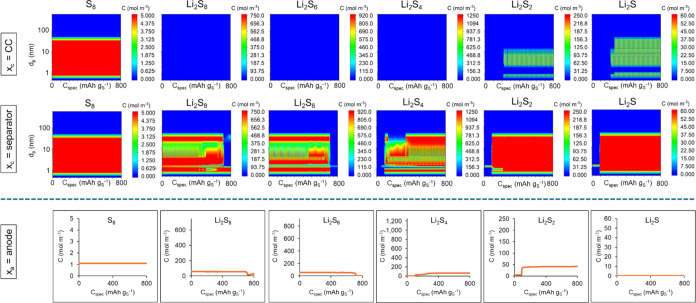
Predicted concentrations of the dissolved sulfur and sulfides in the electrolyte solution as a function of specific capacity during the first discharge of Li–S battery cell with the assumed cathode of 45 wt % S-45 wt % KB-10 wt % PVDF coating: contour plots in cathode as a function of pore size for two different locations (by the current collector and by the separator) and concentration profiles at the anode. The maximum concentration limit in each plot is set to the saturation concentration of that species in the liquid electrolyte.

The same GDC simulation was repeated for a Li–S battery cell without any energy adsorption effects by the binder and no surface redox or Li^+^ intercalation in the binder. This simulation is denoted for a Li–S cell with the assumed cathode of 45 wt % S-45 wt % KB-10 wt % PVDF coating. The predictions yielded a maximum specific capacity of 761 mAh g_S_^–1^ in discharge. Figure 12 presents the predicted concentrations of dissolved sulfur and Li_2_S_*x*_ at the two cathode positions (by the current collector and the separator) and at the anode. Comparing Figure 12 with Figure 11, it can be seen in Figure 12 that the assumed PVDF binder allows for a significantly higher rate of sulfide migration from the cathode by the current collector to the cathode surface by the separator, leaving the negligible amount of Li_2_S_8_, Li_2_S_6_, and Li_2_S_4_ in the cathode by the current collector. This sulfide and sulfur migration continues to the anode with higher concentration profiles of sulfur and sulfides at the anode observed in Figure 12 compared with Figure 11.

## 5 Discussion

The DFT predicted adsorption energies in Figure 6 may be compared to predicted “binding” energy values by MD simulations with regards to the adsorption of Li_2_S_4_ by PSS, *E*_ads_ = 2318 kJ mol^–1^, and by PEDOT, *E*_ads_ = 409 kJ mol^–1^,^46^ where *E*_ads,min_ = −739 kJ mol^–1^ has been predicted for Li_2_S_4_ adsorbed by PEDOT:PSS in the present study. Furthermore, the structures inputted for DFT simulations in the present study are rather simplistic and may not accurately represent the long-range morphology of PEDOT:PSS. This seems to vary depending on the fabrication method, casting, spin coating, spraying, or inkjet printing,^28,30,47,48^ and any annealing. The fabrication method in this study may be considered close to the casting of a thick slurry/paste with solid particles, followed by water evaporation at room temperature without any thermal annealing. Cast PEDOT:PSS films without any thermal annealing exhibit a grain morphology with grain sizes of about 20–30 nm.^49^ A 20 nm layer thickness of the PEDOT:PSS binder around the S-KB particles is estimated in Section 4.1 from Figure 4, which is the same as the optimum PEDOT:PSS particle shell thickness identified in studies of Li–S batteries with a cathode of hollow PEDOT:PSS particles and an internal lining sulfur layer,^50^ where it was found that a thinner PEDOT:PSS shell of 9 nm was insufficient to entrap the soluble polysulfides. Hence, grains of about 20 nm may be assumed in this study, consisting of a central PEDOT-rich core surrounded by a PSS-rich shell, as has been detected in PEDOT:PSS films.^49,51,52^ This may be a reason to argue for even higher adsorption energies for the Li_2_S_*x*_ molecules, with the adsorption being dominated by the outer PSS layer, which exhibits stronger interactions with Li_2_S_*x*_.^46^ However, the dissociation of Li_2_S_*x*_ dissolved in the electrolyte and the migration of S_*x*_^2–^ anions under the influence of the electric field to the counter charged electrode is not modeled by our DFT simulations, which do not consider independent S_*x*_^2–^ anions. Hence, the conservative *E*_ads,ave_ values from the DFT simulations of this study with regard to a simplistic PEDOT:PSS structure may be considered suitable to be employed by the continuum model simulations of the Li–S battery cell in this study.

Given the low diffusion coefficient, *D*_Li^+^,PEDOT:PSS,intercalation_, negligible intercalation of Li^+^ ions has been predicted in the PEDOT:PSS binder nanolayer, even when the lowest possible value of the PEDOT:PSS wall nanolayer *l*_pseudo_ = 3.3 nm was inputted as estimated in Section 4.1 from an assumed homogeneous binder distribution coating the walls of pores greater than 30 nm. Hence, only the surface redox reaction of Li^+^ ions with PEDOT:PSS (*j* = pseudo) is included in the continuum model for the Li–S battery cell.

Figure 7f shows that the hybrid pseudocapacitor-EDLC cell based on 90 wt % KB-PEDOT:PSS electrodes has a low power density for supercapacitor but a good energy density at discharge, with a maximum of 25 Wh kg_electrodes_^–1^ at discharge. This maximum energy density of the hybrid supercapacitor in this study is more than 3 times the maximum energy density of a similar hybrid supercapacitor in a previous study by our group^31^ with Li-ion electrolyte 1 M LiPF_6_ in EC:EMC 50:50 v/v and AC-based coating electrodes with the 10 wt % PEDOT:PSS binder fabricated via the doctor blade technique as in the present study. This demonstrates the superior performance of the KB-based electrodes in the presence of Li-ion electrolyte 1 M LiTFSI in DOL:DME 50:50 v/v, with KB contributing higher specific capacitance and electrical conductivity than AC. Computer simulations of the first GCD cycle of the hybrid pseudocapacitor-EDLC cell of this study revealed that the pseudocapacitive behavior of the PEDOT:PSS binder in the doctor blade-fabricated electrode is due only to the surface redox reaction *j* = pseudo of PEDOT:PSS with attached Li^+^ ions, with negligible contribution of any Li^+^ intercalation in PEDOT:PSS. This is in agreement with the observed CV curves of the similar hybrid pseudocapacitor-EDLC in the Li-ion electrolyte in ref (31), where surface redox was observed in doctor blade-fabricated AC-based electrodes with the PEDOT:PSS binder against intercalation with redox observed in sprayed AC-based electrodes with the PEDOT:PSS binder.

The continuum-level physicochemical models presented in this study are, to the best of our knowledge, the first models of this type incorporating intercalation and surface redox for electrodes with pseudocapacitive binders for supercapacitors and Li–S battery cells. Such enriched continuum multipore models are invaluable tools, as proven in this study, in elucidating mechanisms and the contribution of certain processes during the operation of electrochemical energy storage devices. They can also be employed in the design of electrode materials.

The experimental studies and associated simulations demonstrated the equivalence of the surface redox reaction between Li^+^ ions and the PEDOT:PSS binder at the anode during the hybrid supercapacitor charge and at the cathode of a Li–S cell during discharge. The presence of the PEDOT:PSS binder in the sulfur cathode created a longer first discharge of a specific capacity of 744 mAh g_S_^–1^ in the present experimental study compared with 680 mAh g_S_^–1^ obtained in the first GDC of a similar Li–S cell with the KB-based cathode and the PVDF binder.^21^ Our continuum model simulations confirmed a 7% higher specific capacity in the discharge of a Li–S cell with the PEDOT:PSS binder in the cathode compared to that with the PVDF binder in the cathode, which is due to the term of a surface redox reaction, which takes place in increased rate and changes the GDC slope after 600 mAh g_S_^–1^.

Concerning the effect of the adsorption energy of the PEDOT:PSS binder with respect to sulfur and sulfides, their predicted concentration profiles at the anode in Figure 11 are compared with those in Figure 12, with the latter being the results of a simulation of a Li–S cell with the same 45 wt % S-KB-based cathode but with the PVDF binder in which case the simulations did not include any adsorption effects by the binder. The comparison shows that the PEDOT:PSS binder has reduced the predicted S_8_ concentration at the anode to a third and the concentrations of sulfides at the anode to 1/100th–6/100th to those predicted by the simulation of the Li–S cell with the PVDF binder in the cathode coating. With regard to the adsorption of sulfur and sulfides by the PEDOT:PSS binder, the following effects need to be considered: (a) No binder exists in the micropores and mesopores smaller than 32 nm (interparticle distance), so any sulfides being transported through these pores are not inhibited by PEDOT:PSS. (b) The attraction is strong in the 32 nm interparticle pore, which is assumed to be lined by the PEDOT:PSS to the distance *r*_ads,s_, so the full value of *E*_ads,s_ is applied to all species being transported through this pore. (c) The other three meso- and macropores from the discretized PSD of sizes 52, 82.6, and 600 nm are associated by *E*_ads,s_ values reduced at least 5 times for the 52 nm pore, 17 times for the 82.6 nm pore, and 230 times for the 600 nm pore. Given that the adsorption energy is embedded in an exponential term according to eq 11, an *E*_ads,s_ value reduced by 5 brings an exponential decay factor of 6.7 × 10^–3^, which means that the adsorption effect of the PEDOT:PSS binder is very weak in the pores of 52, 82.6, and 600 nm. However, no sulfur or sulfides are present in the predicted concentrations in the pores of 52, 82.6, and 600 nm in Figures 11 and 12, which means that transport of the dissolved sulfur and sulfides across the cell takes place mainly through the interparticle pore of 32 nm, in which the full strength of *E*_ads,s_ by PEDOT:PSS is applied very effectively.

The continuum model simulations presented in this study might still have some uncertainties. The input PSD of the cathode host was the PSD of the KB powder that was subsequently impregnated with sulfur, the simulation of which resulted in the predicted PSD of the 45 wt %S-KB cathode without taking into account the effect of the binder. The binder would have created a coating of different PSD with reduced total specific volume and area and closed pores with entrapped sulfur in them. The binder distribution on the particle surface controls the fraction of the closed pores from which sulfur cannot escape and the fraction of open channels on the pore surface, allowing the transport of the Li^+^ ions to the sulfur as well as the channel width, which if small of the order of 1 nm or less would prohibit the migration of sulfur and sulfides as the adsorption energy would act fully without any weakening due to the large pore size. In such case, the high adsorption energies by PEDOT:PSS predicted by the DFT simulations, as presented in Figure 6, would substantially reduce the sulfide migration through and away from the cathode, as indeed has been predicted in this study (Figure 11).

## 6 Conclusions

The present study has investigated the mechanisms by which PEDOT:PSS, used as a binder in the cathode, can extend the energy density and improve the performance of Li–S batteries. We have deployed electrochemical testing of Li–S battery cells and supercapacitors with electrodes similar to the cathode host material of the Li–S cells and the same PEDOT:PSS binder, electrolyte, and separator, material characterization at fabrication, and post-mortem after cell cycling, as well as multiscale modeling. The following conclusions have been drawn for a cathode host based on hollow porous carbon particles (KB) and the electrolyte 1 M LiTFSI in DOL/DME:

(a) The PEDOT:PSS binder adsorbs sulfur and polysulfides and inhibits them from migrating to the anode, where the strongest adsorption effect is applied in the interparticle voids where the species are closest to the PEDOT:PSS surface, given the relation between the binder layer thickness and the pore size.
(b) Surface redox between the Li^+^ ions and PEDOT:PSS add pseudocapacitive energy during the discharge of a Li–S battery cell, increasing the specific capacity by 7–9%, as concluded from continuum model-based simulations and experimental studies, respectively. This and the level of the related standard redox potential are consistent with experimental data of the hybrid pseudocapacitor-EDLC based on KB-90 wt % PEDOT:PSS electrodes and the same electrolyte and separator as the Li–S cell.

Further to these conclusions about the beneficial role of the PEDOT:PSS binder for Li–S battery cells, it is realized that depending also on other factors such as the cathode host, separator, and electrolyte, the PEDOT:PSS binder may not fully eliminate the shuttling effect. The proposed multipore continuum model, enhanced with adsorption effects of the pore walls with respect to sulfur and sulfides and also with surface redox effects and validated in the present study, has great potential in being used for the material design of Li–S cells with a liquid electrolyte to assess additional features that may improve Li–S battery performance.
